# Hypoxia’s Impact on Hippocampal Functional Connectivity: Insights from Resting-State fMRI Studies

**DOI:** 10.3390/brainsci15060643

**Published:** 2025-06-14

**Authors:** Julia Micaux, Abir Troudi Habibi, Franck Mauconduit, Marion Noulhiane

**Affiliations:** 1 University Paris-Saclay, CEA, Joliot Institute, NeuroSpin, Clinical and Translational Applied NeuroImaging Research Unit (UNIACT), 91191 Gif-sur-Yvette, France; julia.micaux@outlook.fr (J.M.); abir.habibi@cea.fr (A.T.H.); 2 University of Paris Cité, Inserm, U1141 NeuroDiderot, InDev Team, 75019 Paris, France; 3 CEA, NeuroSpin, CNRS, University of Paris-Saclay, 91191 Gif-sur-Yvette, France; franck.mauconduit@cea.fr

**Keywords:** resting-state functional MRI, cerebral hypoxia, hippocampus, functional connectivity, resting-state networks

## Abstract

The hippocampus is one of the brain’s most vulnerable structures to hypoxia, playing a crucial role in memory and spatial navigation. This sensitivity makes it a key region for understanding the effects of hypoxia on brain connectivity. This review examines the effects of both acute and chronic hypoxia on resting-state networks (RSNs) that contribute to hippocampal functional connectivity (FC). Hypoxia, characterized by a reduced oxygen supply to the brain, can result from environmental factors (such as high-altitude exposure) or hypoxia-induced pathological conditions (including obstructive sleep apnea and hypoxic–ischemic encephalopathy). The hippocampus’s susceptibility to hypoxic damage significantly impairs brain connectivity. This review examines through rs-fMRI studies how hypoxia alters hippocampal FC, focusing on its effects on RSNs involved in hippocampal functions, and compares acute and chronic hypoxic states. We seek to determine whether distinct or shared patterns of FC changes exist between acute and chronic hypoxia, and how hypoxia indirectly changes hippocampal FC, given the challenges of studying it in isolation. By addressing these questions, this review aims to deepen our understanding of hypoxia-induced changes in hippocampal FC and provide insights into potential therapeutic strategies to mitigate its effects on cognitive functions.

## 1. Introduction

### 1.1. Background

A rapid decline in oxygen levels marks the transition from normoxia to hypoxia. Hypoxia can be classified into two main etiology types, acute and chronic, which can arise in both environmental and pathological contexts. Environmental hypoxia, such as high-altitude exposure or voluntary breath-holding in freediving, can either induce transient (acute) or sustained (chronic) oxygen deprivation. Similarly, hypoxia can cause pathological conditions in both acute and chronic forms, including obstructive sleep apnea and hypoxic–ischemic encephalopathy. While these forms differ in etiology, both environmental and pathological hypoxia can lead to significant cognitive and physiological consequences [1,2,3]. Acute hypoxia involves sudden oxygen deprivation, while chronic hypoxia is marked by prolonged exposure to reduced oxygen levels. Both forms can profoundly disrupt hippocampal structure and function, a brain structure involved in memory and spatial navigation [4,5]. The hippocampus is susceptible to oxygen deprivation, making it highly vulnerable to hypoxic injury [6,7,8,9,10]. This vulnerability positions the hippocampus as a critical region for understanding how hypoxia affects neuronal integrity and brain connectivity. Structural MRI studies have shown that both acute and chronic hypoxia can lead to changes in hippocampal volume and neural network disruptions [7,11,12]. Also, disrupted hippocampal function is well documented, leading to cognitive impairments, including memory deficits and executive function decline [4]. These changes likely result from impaired neuronal communication, reduced neurogenesis, and decreased metabolic adaptation [13,14]. Given the hippocampus’s pivotal role in cognitive function, understanding how hypoxia alters its FC is crucial for understanding long-term cognitive effects.

Resting-state functional MRI (rs-fMRI) is a non-invasive imaging technique used to investigate spontaneous brain activity in the absence of task performance. During rs-fMRI acquisition, the subject lies quietly (eyes open or closed) and the scanner captures low-frequency (<0.1 Hz) fluctuations in the blood-oxygen-level-dependent (BOLD) signal across the whole brain. These fluctuations reflect changes in the relative concentrations of oxyhemoglobin and deoxyhemoglobin, serving as an indirect marker of neuronal activity and neurovascular coupling [15,16,17,18,19,20].

Data are typically acquired using gradient-echo echo-planar imaging (EPI) sequences over 5–10 min, providing full-brain coverage with high sensitivity to neurovascular coupling [21]. By using the rs-fMRI technique, researchers can investigate and explore neural and vascular responses to oxygen deprivation, as it provides valuable insights into intrinsic brain connectivity. This method helps identify disruptions in networks, such as the hippocampus and its default mode network (DMN), which are particularly sensitive to hypoxic conditions [22,23,24,25]. Measures such as seed-based connectivity, independent component analysis (ICA), and amplitude of low-frequency fluctuations (ALFF) provide complementary insights into FC and regional spontaneous neural activity [26,27,28]. Advanced tools, including functional connectivity density (FCD) and dynamic causal modeling (DCM), further enhance our understanding of the brain’s functional organization under hypoxic conditions [29,30,31].

However, direct assessments of hippocampal FC during hypoxia have been limited, with most studies focusing on RSN FC and reporting memory loss as a cognitive outcome. This review aims to fill this gap by specifically examining changes in hippocampal FC through RSN alterations under hypoxic conditions.

### 1.2. Scope of Review

This narrative review focuses on environmental hypoxia and hypoxia-induced pathologies, with an emphasis on understanding hippocampal FC changes during such hypoxic conditions. The review aims to achieve the following:

1. Identify the RSNs involved in the hippocampal networks during rest.
2. Explore how rs-fMRI reveals changes in hippocampal FC during hypoxia.
3. Compare the effects of acute and chronic hypoxia on brain FC, examining whether they produce distinct or shared patterns of network changes.

By addressing these questions, this review seeks to provide an integrative perspective on hippocampal adaptability to hypoxic conditions and inform the development of potential interventions to enhance cognitive resilience in individuals exposed to hypoxia. Our objective is not to exhaustively catalogue all published studies on the topic, but rather to synthesize key findings, identify common methodological approaches and limitations, and highlight emerging patterns that may inform future research.

## 2. Physiological Basis of Hypoxia in the Context of Resting-State fMRI

Hypoxia, defined as insufficient oxygen availability to meet metabolic demands, has profound effects on brain physiology and FC [32,33]. Oxygen is critical for ATP production via oxidative phosphorylation. When oxygen levels drop, either suddenly (acute hypoxia) or over prolonged periods (chronic hypoxia), the brain undergoes a cascade of compensatory and pathological processes that can alter neuronal activity and disrupt BOLD signal dynamics [34].

Acute hypoxia triggers rapid oxygen deprivation (e.g., high-altitude or apnea) and elicits immediate systemic responses such as hyperventilation and increased cardiac output. In the brain, it induces oxidative stress and mitochondrial dysfunction, particularly in regions like the hippocampus and prefrontal cortex [35,36]. These changes impair synaptic function and may transiently disrupt resting-state networks, which are sensitive to metabolic alterations. Although some oxidative markers normalize within 24 h, apoptotic pathways may remain active, potentially affecting rs-fMRI readouts beyond the hypoxic exposure window [33,35,37,38].

Chronic hypoxia, characterized by prolonged oxygen deprivation over weeks to years, is commonly associated with conditions such as high-altitude exposure or obstructive sleep apnea, which leads to sustained metabolic stress [34,39]. Adaptations like erythropoiesis and vascular remodeling help maintain oxygen delivery but are often accompanied by white matter changes, hippocampal atrophy, and altered FC patterns in rs-fMRI [40,41]. The structural and functional alterations are also implicated in the cognitive decline observed in chronic hypoxia and may predispose individuals to neurodegenerative diseases such as Alzheimer’s [42]. Moreover, chronic hypoxia impacts hormonal responses, particularly in the context of exercise. For example, chronic resistance training under normobaric hypoxia has been shown to suppress the growth hormone response to exercise in older adults [43]. These findings highlight the complex interplay between chronic hypoxia, systemic physiological responses, and its long-term impact on brain health and cognitive function. Understanding these mechanisms is essential for developing interventions to mitigate the detrimental effects of chronic hypoxia.

**Figure 1:**
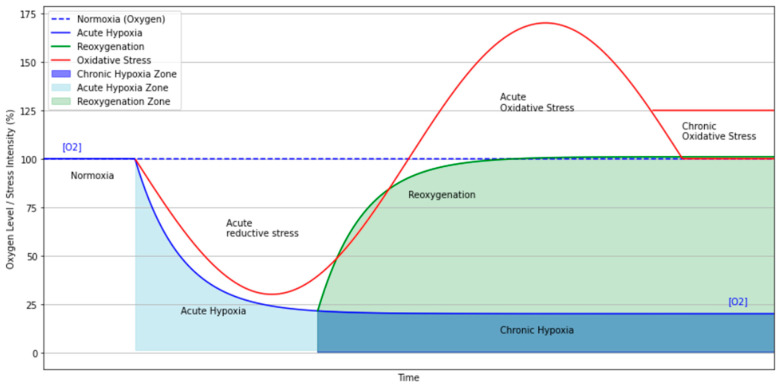
A schematic representation of the physiological phases associated with acute and chronic hypoxia. The transition from normoxia to acute hypoxia triggers a rapid drop in oxygen availability (“acute hypoxia zone”), followed by a reoxygenation phase during which oxidative stress peaks due to a surge in reactive oxygen species (ROS). This phase may transiently alter resting-state network activity through metabolic and neurovascular disruption. If hypoxia persists or if reoxygenation remains incomplete, a state of chronic hypoxia may develop, leading to sustained oxidative stress, neuroinflammation, and structural brain changes. These chronic alterations can disrupt long-range connectivity and modulate baseline BOLD signal fluctuations, as often observed in rs-fMRI studies of patients with chronic hypoxic exposure.

The transition from normoxia to hypoxia and then to reoxygenation (e.g., during intermittent hypoxia or following apnea) initiates dynamic oxidative and metabolic changes that modulate the BOLD signal. As illustrated conceptually in Figure 1, the acute reoxygenation phase is often accompanied by a surge in reactive oxygen species (ROS), which may transiently disrupt local neuronal signaling and induce short-range functional disconnection or altered network synchrony detectable by rs-fMRI [25,37]. If oxidative stress persists, it may evolve into a chronic state. This may progressively affect baseline neuronal activity and contribute to disruptions in long-range connectivity [35]. These alterations may reflect broader shifts in brain network organization associated with sustained metabolic stress and neurovascular adaptations.

By understanding how oxygen availability shapes brain physiology, rs-fMRI can serve as a valuable tool for capturing both transient and lasting effects of hypoxia on neural networks.

## 3. Hippocampal Functional Networks at Rest

Rs-fMRI reveals intrinsic brain organization through resting-state networks (RSNs), which reflect patterns of spontaneous, coordinated activity across different brain regions [25,44]. The hippocampus, a key hub for memory, spatial navigation, and future thinking, is functionally integrated into multiple RSNs, several of which are particularly relevant for understanding how hypoxia may impact brain function.

The DMN, first identified by Greicius et al. [45], is closely linked to the hippocampus, is active during self-referential and memory-related processes, and is especially sensitive to metabolic and vascular changes, making it a primary target of hypoxia-related dysfunction [46,47,48,49,50]. The fronto-parietal network (FPN), involved in executive control, attention, and cognitive flexibility, modulates hippocampal activity during memory encoding and retrieval, facilitating the dynamic balance between internal and goal-directed cognition [51].

The salience network supports the detection of emotionally or contextually significant stimuli, as its interactions with the hippocampus help prioritize memories based on relevance and emotional salience [52,53,54].

The central executive network (CEN) engages in higher-order cognitive functions, such as working memory, decision-making, and problem-solving. It enables cognitive control and is activated during tasks requiring sustained attention or complex reasoning. The hippocampus interacts with the CEN to ensure smooth transitions between memory processes, cognitive control, and emotional regulation [55,56].

Attention-related networks, the dorsal (DAN) and ventral VAN attention networks, coordinate goal-directed and stimulus-driven attention, respectively [57,58]. The hippocampus contributes contextual and spatial information essential for sustaining attention and guiding behavior [59,60,61,62]. Sensory–motor networks (SMNs), while primarily responsible for sensory processing and motor control, also interact with hippocampal subregions such as the dentate gyrus and CA3 during spatial navigation and sensorimotor integration [49,63].

Lastly, the visual network, encompassing primary and extra-striate visual areas, engages with the hippocampus during visuo-spatial tasks, supporting memory-guided perception and navigation [50,52,64].

**Figure 2:**
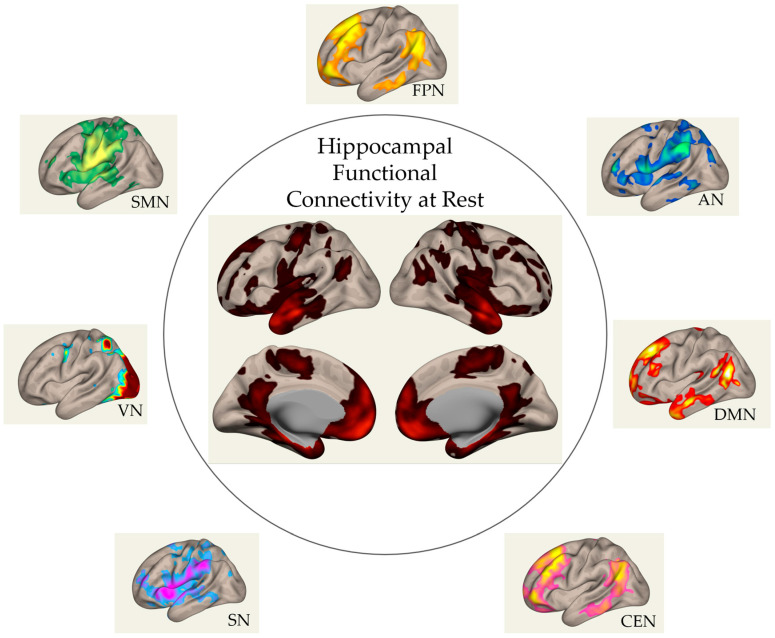
Surface plots of the FC of the hippocampal network at rest with other RSNs identified using a seed-based connectivity analysis on a sample of 20 healthy adult males from the HipoXia Project (ANR-21-CE37-0022 HippoXia, Ethic Approval Number: 2022-A01030-43). DMN: default mode network; SMN: sensory–motor network; SN: salience network; CEN: central executive network; VN: visual network; AN: attention network; FPN: fronto-parietal network.

Together, these interactions highlight the hippocampus’s central role in a broad range of functional networks, as presented in Figure 2. As such, any hypoxia-induced disruption in these RSNs, whether acute or chronic, is likely to alter hippocampal FC. This hypothesis will be further explored in the following sections.

## 4. Rs-fMRI in Acute Hypoxia

### 4.1. Cerebral Adaptations to Hypoxic Environments

Hypoxic environments, particularly those causing acute hypoxia, significantly challenge brain function by limiting oxygen supply to neuronal tissues. Such conditions can arise in high-altitude environments, during intense physical activity, breath-holding, aviation, carbon monoxide poisoning, or in confined spaces with low oxygen levels.

Short-term exposure leads to functional changes in the hippocampus due to its high sensitivity to oxygen levels. Functional recordings showed a significant increase in the frequency of slow oscillations (2.1–2.2 Hz) under hypoxic conditions. Additionally, the firing frequency of interneurons in the hilus and CA3 region decreased, while the activity of pyramidal cells in the CA1 and CA3 regions increased. Notably, hypoxia also disrupted the regularity of neuronal firing patterns in these regions. The findings indicate that mild, short-term hypoxia can induce significant alterations in hippocampal network activity, which may affect information processing [12].

At high altitude, the body engages compensatory mechanisms such as increased ventilation, elevated heart rate, and augmented erythropoiesis [65]. Yet these physiological responses are often insufficient to safeguard optimal hippocampal function.

Recent studies using rs-fMRI have demonstrated that acute high-altitude exposure leads to decreased FC between the hippocampus and critical cortical regions, including the prefrontal cortex and the DMN. This reduced FC correlates with deficits in sustained attention and memory retrieval, highlighting the hippocampus as a central hub impaired during hypoxic stress [66].

Additionally, simulated high-altitude hypoxia has shown decreased FC between important networks linked to the hippocampus, such as the FPN and visual network, as well as the DAN and VAN, leading to perceptual deficits across sensory modalities, notably auditory and visual processing [22]. These connectivity disruptions manifest as longer reaction times, reduced response accuracy, impaired memory, and overall cognitive slowing, particularly in prefrontal and parietal cortices [66,67].

Interestingly, controlled breathing techniques have emerged as potential modulators of these effects by improving cerebral oxygenation and supporting hippocampal activity under acute hypoxia [68]. In aviation, pilots are exposed to rapid altitude ascents, which have been associated with hippocampal volume alterations and shifts in DMN connectivity, possibly reflecting adaptive changes [69,70]. However, exposure to aircraft noise has been linked to hippocampal dysfunction and related cognitive impairments, especially in working memory and emotional regulation, with changes observed in connectivity involving the amygdala, thalamus, and frontal gyrus [71,72,73].

In contrast, hyperoxia conditions have been shown to enhance connectivity within the DMN and AN, reinforcing the hippocampus’s sensitivity to oxygen level fluctuations [5,74].

Despite the detrimental effects of acute hypoxia, the brain demonstrates a remarkable capacity for adaptation. With repeated exposure or acclimatization, cognitive functions such as attention and executive control may be preserved or even improved [75]. This is attributed to neuroplastic mechanisms, where the brain compensates for reduced connectivity by enhancing activity in alternative regions and responses in specific regions [76,77,78]. Yet, although such compensatory strategies help mitigate some effects, they cannot fully restore hippocampal-dependent processes like flexible memory encoding and retrieval [79].

**Figure 3:**
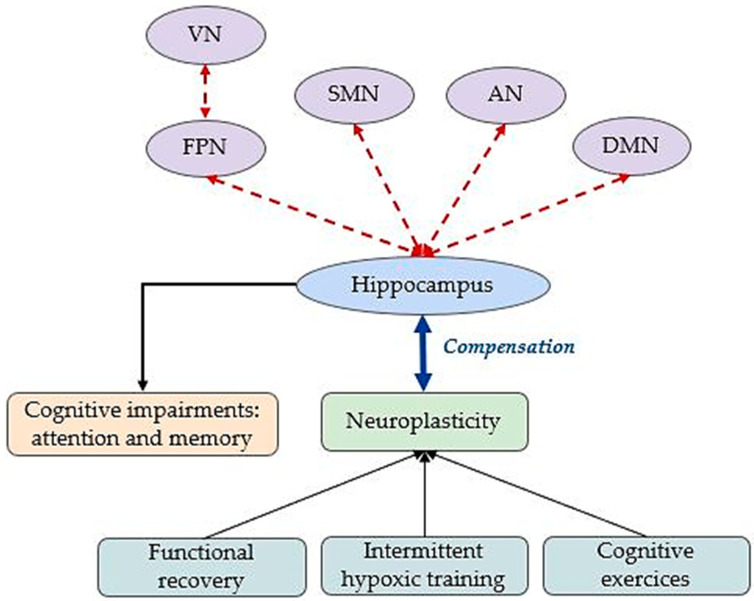
Cerebral adaptations to environmental acute hypoxia. The red dashed edges illustrate altered FC. DMN: default mode network; SMN: sensory–motor network; VN: visual network; AN: attention network; FPN: fronto-parietal network.

Lastly, interventions such as intermittent hypoxic training and cognitive stimulation were shown to promote neuroplasticity, enhance hippocampal resilience, and activate neuroprotective pathways, offering promising strategies to counteract the cognitive challenges associated with hypoxia [77]. Figure 3 illustrates hippocampal FC changes under environmental acute hypoxia.

### 4.2. Cerebral Adaptations to Hypoxia-Induced Pathological Conditions

Acute hypoxia, as seen in conditions like hypoxic–ischemic encephalopathy (HIE) and acute mountain sickness (AMS), induces significant cerebral changes, particularly affecting the hippocampus. Despite compensatory neurophysiological mechanisms, these adaptations often fail to fully maintain homeostasis under low-oxygen conditions, contributing to persistent altitude-related health issues such as AMS [80].

In AMS, rapid ascent to HA leads to brain-centered symptoms, with rs-fMRI having identified the SMN as a key predictor of AMS severity and its cognitive [81]. Importantly, hypoxia-related disturbances prominently affect the hippocampus, a region susceptible to oxygen deprivation, resulting in disruptions across connected networks such as the DMN and FPN. These network alterations impair attentional performance under hypobaric hypoxia, exacerbated by additional environmental stressors such as low temperatures, dehydration, and sleep deprivation [82].

Similarly, in the HIE, a leading cause of long-term neurodevelopmental deficits, the hippocampus is particularly vulnerable to hypoxic injury. Severe HIE cases show decreased local network efficiency and altered connectivity, notably involving the left rolandic operculum and superior temporal gyrus [83]. FC reductions within sensory–motor and cognitive networks have been reported in moderate to severe HIE [84], while compensatory increases between motor areas and frontal, temporal, and parietal cortices suggest attempts at functional reorganization [85].

The hippocampus, through its integrative role between memory and sensorimotor processes, appears central in these adaptive mechanisms. Its compromised function not only underlies cognitive impairments but also predicts long-term neurodevelopmental outcomes. Indeed, network degradation, particularly affecting hippocampal connections, correlates with poorer motor and developmental scores [86].

Despite structural vulnerabilities, evidence of neuroplasticity offers promising perspectives: cooled children with HIE without severe disabilities exhibit functional recovery, hinting at the hippocampus’s capacity for adaptive reorganization [87]. Similarly, animal models confirm spontaneous hippocampal plasticity supporting motor recovery following neonatal hypoxic injury [88].

Moreover, studies focusing on full-term infants with varying severity of HIE show preserved small-world network organization, but severe cases present reduced local efficiency and fewer hub regions, notably involving hippocampal structures, which could serve as early biomarkers for targeted interventions [83].

**Figure 4:**
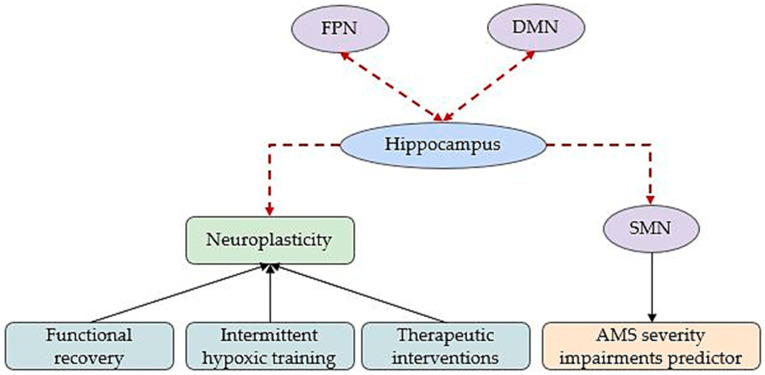
Cerebral adaptations to acute hypoxia-induced pathologies. Dashed edges illustrate altered FC. DMN: default mode network; SMN: sensory–motor network; FPN: fronto-parietal network; AMS: acute mountain sickness.

As shown in Figure 4, the hippocampus emerges as a central node mediating neuroplastic responses and predicting cognitive and motor outcomes, making it a critical target for therapeutic strategies such as intermittent hypoxic training, neuroprotective interventions, and rehabilitation aimed at enhancing functional recovery.

## 5. Rs-fMRI in Chronic Hypoxia

### 5.1. Cerebral Adaptations to Chronic Hypoxic Environments

Prolonged exposure to high-altitude environments, where oxygen levels are significantly reduced, has profound effects on cognitive functions, particularly affecting the hippocampus. Chronic hypoxia leads to reduced FC between the hippocampus and other brain regions involved in memory processes [89], contributing to lasting cognitive deficits, especially in memory formation and spatial navigation [90].

Adaptations to high-altitude environments are evident in populations like Tibetans, who show structural changes in the hippocampus, including the bilateral CA3, right CA4, and dentate gyrus. These adaptations are potentially linked to genetic factors influencing hemoglobin and hematocrit levels, which support oxygen transport [91]. In contrast, Han Chinese individuals living at high altitudes showed smaller hippocampal volumes compared to those living at sea level, though the differences were not always statistically significant [92]. The effect of high-altitude exposure on cognitive abilities varies based on altitude and duration of exposure, with psychomotor skills and long-term memory showing the most pronounced decline, while perceptual processes, inhibitory control, and problem-solving abilities often remain unaffected [93,94].

Additionally, high-altitude exposure impairs verbal working memory and brain functionality [95], with decreased FC post exposure strongly correlated with declines in memory and reaction time. These FC changes reflect alterations in the topological properties of brain functional networks, particularly in regions involved in attention, perception, memory, and motor modulation, ultimately impairing cognitive performance. In particular, volume changes in the left hippocampus and right caudate were positively associated with improvements in delayed verbal memory performance [96,97]. As the hippocampus plays a critical role in recollection, damage to this medial temporal structure leads to selective deficits in this function. Specifically, the left hippocampus is more involved than the right in cognitive processes such as temporal sequence memory [98], associative memory for match–mismatch tasks [99], and event memory [100].

Chronic hypoxia further disrupts neuronal activity and FC in the visual network, leading to increased activity in areas such as the right calcarine gyrus and supplementary motor cortex, while reducing connectivity between the lingual gyrus and postcentral gyrus, indicating SMN disruption [101], which could be a crucial predictor for ACM [81]. Executive function is similarly impaired, with decreased accuracy and slower response times in cognitive tests linked to reduced gray matter density in the olfactory cortex, insula, and temporal lobes [102]. Despite these adaptive brain activity changes in response to low oxygen, long-term exposure may have detrimental effects, with FC alterations correlating with cognitive impairments in the FPN and SMN [103]. Patients with CMS and soldiers exposed to high altitudes also exhibit significant brain structure and functional changes, with CMS patients showing memory decline and soldiers demonstrating increased FC as a compensatory mechanism [104,105]. These findings suggest that chronic hypoxia induces brain reorganization and neural compensation [89,106,107,108]. Animal models further support these effects, with rats exposed to prolonged hypobaric hypoxia showing impaired cognitive performance [109], structural abnormalities, neural apoptosis, and mitochondrial damage in the hippocampus and frontal cortex [110]. Furthermore, cognitive deficits have been observed in mice exposed to a simulated high altitude, particularly in learning and memory tasks [111].

**Figure 5:**
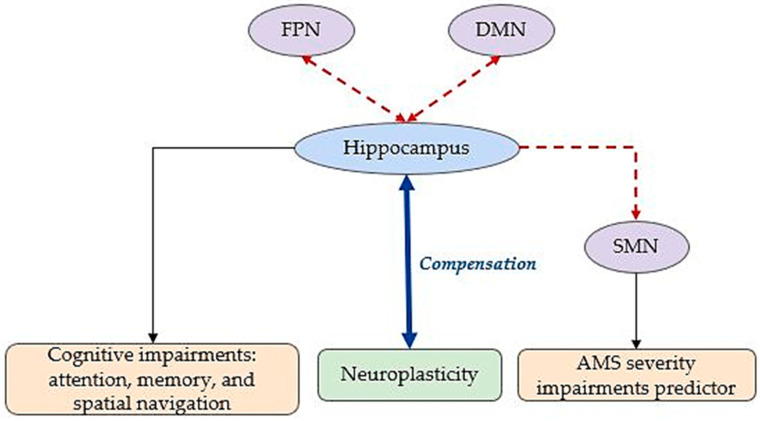
Cerebral adaptations to environmental chronic hypoxia. The red dashed edges illustrate altered FC. DMN: default mode network; SMN: sensory–motor network; FPN: fronto-parietal network.

Overall, chronic exposure to high-altitude environments significantly alters hippocampal functional integrity and structure, as illustrated in Figure 5, leading to cognitive impairments. These insights emphasize the need for strategies to mitigate the long-term effects of hypoxia on brain health [112].

### 5.2. Cerebral Adaptations to Hypoxia Induced by Pathological Conditions

Obstructive sleep apnea (OSA) is characterized by repeated breathing interruptions during sleep, leading to fragmented sleep and intermittent hypoxia, which has been associated with cognitive deficits, particularly in attention, memory, and executive function [113]. Studies suggest that OSA can affect crucial brain structures such as the hippocampus and frontal cortex, which are integral for cognitive performance [114].

FC in the DMN has been significantly associated with hypoxemia measures, such as the apnea–hypopnea index (AHI) and oxygen desaturation index (ODI) [115], suggesting that disrupted connectivity in brain regions, especially in the hippocampus, correlates with poorer cognitive outcomes. Patients with OSA exhibit altered brain connectivity, particularly involving the hippocampus. These changes have been linked to intermittent nocturnal hypoxia and sleep fragmentation, both of which are hallmark features of OSA. While some studies suggest a compensatory mechanism in certain populations, such as Tibetans with OSA [116], the cumulative impact of repeated hypoxic episodes and disrupted sleep architecture may impair hippocampal function through complementary pathways, including neuroinflammation, oxidative stress, and reduced neurogenesis [117].

Further research has shown functional differences under high-altitude conditions in the hippocampus, reporting that hypoxia and disrupted sleep could contribute to neuronal loss in the hippocampus [118]. Further animal studies have shown that prolonged sleep deprivation significantly suppresses cell proliferation in the dentate gyrus of the hippocampus, with no evidence of recovery after a short period of sleep. Both sleep-deprived rats and those given a brief recovery sleep showed a marked reduction in new cell production compared to controls, with the effect being more pronounced in the posterior hippocampus. These findings suggest that extended sleep loss has a lasting negative impact on hippocampal neurogenesis that is not quickly reversed [119].

Dynamic FC (dFC) studies have also highlighted changes in FC related to sleep indicators and cognitive performance, with altered FC in networks such as the DMN, DAN, VAN, and SN contributing to neuropsychological deficits [120,121,122].

Abnormal dynamic regional spontaneous brain activity has been observed in patients with OSA, especially in the frontal gyrus, cingulate gyrus, and precuneus [121].

OSA patients with MCI showed altered FC in various brain regions such as the temporal gyrus, the frontal gyrus, and the bilateral posterior cingulate/calcarine/cerebellar anterior lobe [122]. Given that these regions, especially the frontal gyrus, are functionally connected with the hippocampus, it is plausible that FC changes in OSA could indirectly impact hippocampal connectivity. However, this hypothesis has not yet been directly confirmed by the available studies.

Additionally, topological analysis in severe, untreated OSA patients revealed disrupted small-world network properties, with decreased global efficiency and increased local efficiency, further suggesting a potential mechanism for cognitive impairment in OSA [123]. Spontaneous brain activity analysis has shown that OSA patients exhibit lower FC in regions associated with the DMN, while showing higher FC in regions such as the cerebellum, cingulate gyrus, and lentiform nucleus, with these changes correlating negatively with sleep stages [124]. In older adults with OSA, increased FC between hippocampal regions, such as the para-hippocampal cortex, correlates with severe nocturnal hypoxemia and poorer working memory performance [125]. Similar cognitive impairments are seen in children with OSA, where changes in brain activity and FC measures highlight the impact of hypoxia on hippocampal function [126].

Chronic environmental hypoxia, as seen in chronic mountain sickness (CMS), similarly affects brain function [80], with individuals suffering from CMS exhibiting abnormal brain activity across multiple regions, particularly the para-hippocampal gyrus [127]. This suggests that prolonged exposure to chronic hypoxia enhances neuronal activity and enlarges hippocampal volume, which in turn disrupts the normal physiological regulation of hyperventilation, ultimately exacerbating the hypoxic condition as reported by other studies [128,129,130].

**Figure 6:**
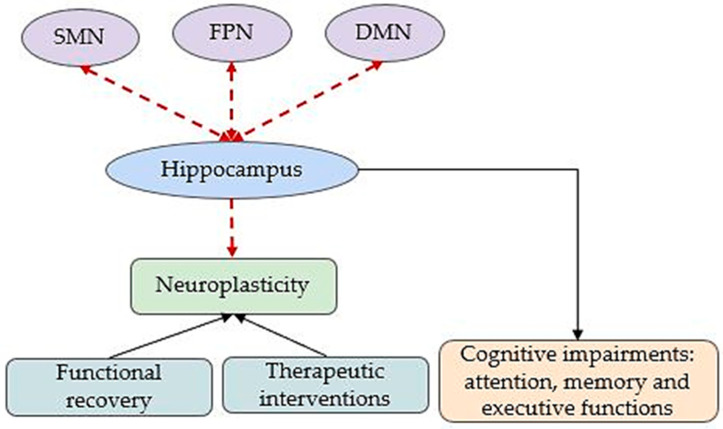
Cerebral adaptations to pathological conditions induced by chronic hypoxia. The red dashed edges illustrate altered FC. DMN: default mode network; SMN: sensory–motor networks; FPN: fronto-parietal network.

Given the heterogeneity of the available findings and the limited number of studies specifically investigating the effects of chronic hypoxia on the hippocampus, we summarized in Figure 6 the main cerebral changes reported in chronic hypoxia-induced pathologies. These changes involve altered FC between the SMN, FPN, and DMN with the hippocampus, which may contribute both to cognitive impairments and to disrupted neuroplasticity necessary for functional recovery.

## 6. Acute and Chronic Hypoxia Patterns

As summarized in Table 1, acute and chronic environmental hypoxia both impact brain FC, particularly in networks involving the hippocampus, which plays a crucial role in memory and cognitive control. In both conditions, studies consistently report reduced hippocampal FC with regions such as the prefrontal cortex and the DMN, which correlates with impairments in memory retrieval and executive functions [66,71].

However, the patterns of disruption and adaptation differ between acute and chronic hypoxia. Acute hypoxia elicits immediate physiological responses (e.g., increased ventilation and heart rate) aimed at enhancing oxygen delivery. The responses often fall short, leading to short-term cognitive deficits and transient FC alterations [78]. Conversely, chronic environmental hypoxia induces long-term structural and functional brain changes, including persistent reductions in hippocampal FC. These adaptations involve complex genetic and environmental mechanisms, such as increased hemoglobin, which may partially compensate for oxygen deprivation, but do not fully prevent long-term cognitive decline [77,101].

Similarly, acute and chronic pathological hypoxia both affect brain FC with similar network targets but distinct trajectories. Acute pathological hypoxia leads to immediate, severe disruptions in brain activity, resulting in rapid cognitive impairment. Yet there is potential for recovery through neuroplasticity, particularly with therapeutic interventions [85,87]. Chronic pathological hypoxia results in long-term, intermittent, and cumulative FC disruptions, particularly in the hippocampus and attentional networks. While some compensatory increases in FC have been observed, they are often insufficient to fully mitigate the sustained cognitive impairments [89,110].

In summary, both acute and chronic pathological hypoxia impair cognitive function and brain connectivity, especially in the hippocampus. Acute hypoxia tends to cause immediate but potentially reversible effects, whereas chronic hypoxia produces more durable impairments with limited compensatory recovery.

## 7. Discussion

Despite growing interest in the impact of hypoxia on brain function, hippocampal FC under hypoxic conditions remains insufficiently characterized, even though the hippocampus is known to be highly vulnerable to oxygen deprivation [8,131]. This narrative review aims to shed light on the functional changes within the hippocampal network by examining FC changes in RSNs associated with the hippocampus, focusing on both environmental and pathological hypoxia. We highlighted (1) RSNs associated with the hippocampal network during rest, (2) how rs-fMRI uncovers changes in hippocampal connectivity under hypoxic conditions, and (3) the effects of acute and chronic hypoxia on brain connectivity, analyzing whether these conditions yield distinct or overlapping patterns of network changes.

At rest, the hippocampus is primarily embedded with the DMN, but it also interacts with networks involved in memory, executive function, and attention. Several rs-fMRI studies have shown that acute hypoxia, marked by a sudden drop in oxygen availability, leads to transient disruptions in FC between the hippocampus and cortical areas, notably in the DMN and attention network, with accompanying cognitive impairments such as slower reaction times and memory deficits [22]. In contrast, chronic hypoxia, often due to sustained environmental or induced pathological conditions such as high-altitude exposure or OSA, induces more persistent and widespread FC alterations. These include long-term reductions in FC between the hippocampus and regions critical for memory and spatial processing, along with potential but often insufficient compensatory increases in FC elsewhere [132]. Overall, chronic hypoxia, in both environmental hypoxia and induced pathologies, may lead to adaptive neuroplastic changes, such as compensatory increases in connectivity in certain brain areas, although these compensatory mechanisms are often insufficient to prevent cognitive decline.

Despite these differences in temporal profiles between acute and chronic hypoxia, both forms consistently disrupt FC patterns, particularly involving hippocampal interactions with memory and executive-related brain networks. However, comparing findings across studies remains challenging due to significant methodological heterogeneity. Differences in hypoxia-induced protocols (e.g., simulated altitude, clinical OSA), subject populations (e.g., age, comorbidities), rs-fMRI acquisition parameters (e.g., scan length, TR), and analytic strategies (e.g., seed-based vs. data-driven approaches) can lead to inconsistent results and hinder metanalytic comparisons [133,134,135].

A promising methodological advance in recent years is the integration of rs-fMRI with arterial spin labeling (ASL) perfusion imaging, allowing for simultaneous investigation of both functional and physiological changes. Unlike rs-fMRI alone, which is sensitive to neurovascular fluctuations without direct metabolic quantification, ASL provides quantitative measures of cerebral blood flow (CBF) and, in more advanced implementations, oxygen extraction and metabolism. Combined ASL-rs-fMRI approaches have been used to fit biophysical models estimating the cerebral metabolic rate of oxygen consumption (CMRO_2_), thereby offering a more complete picture of tissue oxygenation and neurovascular coupling under hypoxic stress [136,137,138].

One notable technique includes dual-echo ASL acquisitions, which allows researchers to obtain both perfusion and BOLD data without prolonging scan duration. These data can be fitted to flow metabolism matching models to estimate voxel-wise CMRO_2_ [139]. Additionally, early attempts at single-sequence acquisitions, such as the EPI-ASL-PAIR sequence, have demonstrated feasibility for integrating ASL and BOLD contrasts in a resting-state context within a short 5 min scan per slice [140]. However, most CMRO_2_ quantification methods were originally developed for task-based studies and are yet to be validated robustly in the resting-state paradigm [141].

Despite their promise, these multimodal techniques introduce additional complexity, require careful calibration, and often demand specialized MRI hardware. To date, there is no widely accepted standard for integrating ASL and rs-fMRI within a single, harmonized acquisition and analysis pipeline [142]. While both ASL and rs-fMRI rely on BOLD contrast mechanisms, they probe different components of the hemodynamic response: ASL primarily captures CBF and perfusion dynamics, while rs-fMRI reflects low-frequency oscillations related to neural synchrony. Their partial complementarity offers a compelling rationale for joint acquisition, but interpretation requires careful modeling of neurovascular coupling [143].

To fully realize the clinical potential of these advanced imaging techniques in hypoxia research, several critical steps are needed: (1) standardization of multimodal imaging protocols; (2) extending CMRO_2_ quantification to resting-state paradigms (current methods for voxel-wise CMRO_2_ estimation need to be adapted and validated for resting-state data, especially under spontaneous physiological fluctuations induced by hypoxia); (3) integration with cognitive and behavioral metrics; and finally (4) validation in prospective clinical models for monitoring hypoxia-related neurological disorders.

## 8. Conclusions

In conclusion, this review highlights the critical yet underexplored area of hippocampal FC changes under hypoxic conditions, emphasizing both acute and chronic environmental and pathological hypoxia. The hippocampus, due to its high metabolic demand, emerges as a key structure vulnerable to oxygen deprivation, with acute hypoxia associated with transient but potentially reversible FC disruptions, and chronic hypoxia linked to enduring structural and functional impairments.

Beyond identifying these connectivity patterns, we underscore the added value of advanced multimodal approaches, particularly the integration of rs-fMRI with ASL perfusion imaging. These techniques offer complementary insights into both neural and vascular dimensions of brain function and hold promise for improving the specificity of hypoxia-related biomarkers.

Nonetheless, to translate these developments into clinically useful tools, several challenges remain. Chief among them is the lack of standardization across imaging protocols, analysis pipelines, and outcome metrics. We therefore advocate coordinated efforts to harmonize acquisition parameters, validate multimodal biomarkers in prospective cohorts, and link imaging findings to cognitive and behavioral outcomes. Addressing these methodological and translational gaps will be essential for advancing our understanding of hypoxia-induced hippocampal dysfunction and for informing targeted therapeutic interventions.

## Figures and Tables

**Table 1:** Summary of key findings on effects of acute and chronic hypoxia on hippocampal functional connectivity.

Hypoxia’s Type	Key Findings	Affected Networks
Acute Hypoxia (e.g., High Altitude, Aviation)	Decreased FC between the hippocampus and prefrontal cortex/DMN: impaired attention and memory.	DMN, FPN, DAN, VAN, Visual, Prefrontal
	Disrupted FC in DAN, VAN, and FPN: longer reaction times.	
	Controlled breathing improves cerebral oxygenation.	
	Aviation-related hypoxia alters hippocampal volume and DMN connectivity.	
Pathological Acute Hypoxia (e.g., HIE, AMS)	FC impairments in DMN, SMN, and FPN, with SMN as a key predictor of AMS severity.	DMN, SMN, FPN, Motor, Temporal
	HIE shows decreased local efficiency and hippocampal connectivity.	
	Compensatory increases in FC among motor, frontal, and parietal areas: neuroplasticity observed in recovery cases.	
Chronic Environmental Hypoxia (e.g., High Altitude)	Reduced hippocampal FC with memory networks: cognitive decline in memory and spatial navigation.	DMN, FPN, SMN, Visual, Memory Networks
	FC disruptions in visual network and SMN: adaptation varies across populations.	
	Animal models show hippocampal damage and mitochondrial impairment.	
Chronic Pathological Hypoxia (e.g., OSA, CMS)	Altered FC in DMN, DAN, VAN, and SN; hippocampal dysfunction linked to intermittent hypoxia and sleep fragmentation: cognitive impairments in attention, memory, and executive functions.	DMN, VAN, DAN, SN, Frontal, Para-Hippocampal Gyrus
	Topological disruptions in network efficiency.	
	Para-hippocampal activity changes in CMS; observed effects in adults, children, and animal models.	

## Data Availability

The datasets referenced in this article are not publicly available, as participants consented to their data being used solely under the supervision of the principal investigator. Requests to access the datasets should be directed to M.N.

## Abbreviations

The following abbreviations are used in this manuscript:

AHI	Apnea–Hypopnea Index
ALFF	Amplitude of Low-Frequency Fluctuations
AMS	Acute Mountain Sickness
ASL	Arterial Spin Labeling
ATP	Adenosine Triphosphate
BH	Breath Holding
BOLD	Blood-Oxygen-Level Dependent
CEN	Central Executive Network
CMRO_2_	Cerebral Metabolic Rate of Oxygen
CMS	Chronic Mountain Sickness
DAN	Dorsal Attention Network
DCM	Dynamic Causal Modeling
dFC	Dynamic Functional Connectivity
DMN	Default Mode Network
EPI	Echo-Planar Imaging
fALFF	Fractional Amplitude of Low-Frequency Fluctuation
FC	Functional Connectivity
FCD	Functional Connectivity Density
FPN	Fronto-Parietal Network
HA	High Altitude
HIE	Hypoxic–Ischemic Encephalopathy
ICA	Independent Component Analysis
MCI	Mild Cognitive Impairment
ODI	Oxygen Desaturation Index
OSA	Obstructive Sleep Apnea
PAIR	Presaturation with Inversion Recovery
ReHo	Regional Homogeneity
ROS	Reactive Oxygen Species
rs-fMRI	Resting-State Dunctional Magnetic Resonance Imaging
RSN	Resting-State Network
SMN	Sensory–Motor Network
SN	Salience Network
VAN	Ventral Attention Network
VN	Visual Network

## References

[1] West J.B. (2012). Respiratory Physiology: The Essentials.

[2] Davis C., Hackett P. (2017). Advances in the prevention and treatment of High-Altitude Illness. Emerg. Med. Clin..

[3] Kumar V., Abbas A.K., Aster J.C. (2017). Robbins Basic Pathology E-Book.

[4] Choudhry H., Harris A.L. (2018). Advances in Hypoxia-Inducible Factors Biology. Cell Metab..

[5] Wu Y.W., Tang C.Y., Ng J., Wong E., Carpenter D., Tao X. (2014). Effects of hyperoxia on resting state functional magnetic resonance imaging. NeuroReport.

[6] Kawasaki K., Traynelis S.F., Dingledine R. (1990). Different responses of CA1 and CA3 regions to hypoxia in rat hippocampal slice. J. Neurophysiol.

[7] Hencz A.J., Magony A., Thomas C., Kovacs K., Szilagy G., Pal J., Sik A.B. (2024). Sort-term hyperoxia-induced functional and morphological changes in rat hippocampus. Front. Cell. Neurosci..

[8] Lana D., Ugolini F., Giovannini M.G. (2020). An Overview on the Differential Interplay Among Neurons–Astrocytes–Microglia in CA1 and CA3 Hippocampus in Hypoxia/Ischemia. Front. Cell. Neurosci..

[9] McClelland J.L., Pribram K.H. (2018). Role of the hippocampus in Learning and memory: A Computational Analysis. Brain and Values.

[10] Zhu Y. (2019). Emotion Regulation of Hippocampus Using Real-Time fMRI Neurofeedback in Healthy Human. Front. Hum. Neurosci..

[11] Wang Z.-X. (2022). Changes in Hippocampus and Amygdala Volume with Hypoxic Stress Related to Cardiorespiratory Fitness under a High-Altitude Environment. Brain Sci..

[12] Hencz A., Magony A., Thomas C., Kovacs K., Szilagyi G., Pal J., Sik A. (2023). Mild hypoxia-induced structural and functional changes of the hippocampal network. Front. Cell. Neurosci..

[13] Aboouf M.A., Thiersch M., Soliz J., Gassmann M., Schneider Gasser E.M. (2023). The Brain at High Altitude: From Molecular Signaling to Cognitive Performance. Int. J. Mol. Sci..

[14] Cui C. (2024). Cerebral Hypoxia-Induced Molecular Alterations and Their Impact on the Physiology of Neurons and Dendritic Spines: A Comprehensive Review. Cell. Mol. Neurobiol..

[15] Drew P.J. (2019). Vascular and neural basis of the BOLD signal. Curr. Opin. Neurobiol..

[16] Kim S.-G., Ogawa S. (2012). Biophysical and Physiological Origins of Blood Oxygenation Level-Dependent fMRI Signals. J. Cereb. Blood Flow Metab..

[17] Uludag K., Uludag K., Ugurbil K., Berliner L. (2015). Physiology and Physics of the fMRI Signal. fMRI: From Nuclear Spins to Brain Functions.

[18] Bandettini P.A. (2012). Twenty years of functional MRI: The science and the stories. NeuroImage.

[19] Yousaf T., Dervenoulas G., Politis M., Politis M. (2018). Chapter Two—Advances in MRI Methodology. Imaging in Movement Disorders: Imaging Methodology and Applications in Parkinson’s Disease.

[20] Bijsterbosch J., Smith S.M., Beckmann C. (2017). An Introduction to Resting State FMRI Functional Connectivity.

[21] Lv H. (2018). Resting-State Functional MRI: Everything That Nonexperts Have Always Wanted to Know. Am. J. Neuroradiol..

[22] Liu J. (2022). Impaired brain networks functional connectivity after acute mild hypoxia. Medicine.

[23] Fox M.D., Raichle M.E. (2007). Spontaneous fluctuations in brain activity observed with functional magnetic resonance imaging. Nat. Rev. Neurosci..

[24] Zuo X.-N., Xing X.-X. (2014). Test-retest reliabilities of resting-state FMRI measurements in human brain functional connectomics: A systems neuroscience perspective. Neurosci. Biobehav. Rev..

[25] Smitha K.A., Akhil Raja K., Arun K.M., Rajesh P.G., Thomas B., Kapilamoorthy T.R., Kesavadas C. (2017). Resting state fMRI: A review on methods in resting state connectivity analysis and resting state networks. Neuroradiol. J..

[26] Lurie D.J., Kessler D., Basset D.S., Betzel R.F., Breakspear M., Kheilholz S., Kucyi A., Liégeois R., Lindquist M.A., McIntosh A.R. (2020). Questions and controversies in the study of time-varying functional connectivity in resting fMRI. Netw. Neurosci..

[27] Lee T.-W., Lee T.-W. (1998). Independent Component Analysis. Independent Component Analysis: Theory and Applications.

[28] Yang H. (2007). Amplitude of low frequency fluctuation within visual areas revealed by resting-state functional MRI. NeuroImage.

[29] Tomasi D., Volkow N.D. (2010). Functional connectivity density mapping. Proc. Natl. Acad. Sci. USA.

[30] Friston K.J., Kahan J., Biswal B., Razi A. (2014). A DCM for resting state fMRI. NeuroImage.

[31] Medaglia J.D. (2017). Graph Theoretic Analysis of Resting Stae Functional MR Imaging. Neuroimaging Clin. N. Am..

[32] Foltyn J., Ploszczyca K., Czuba M., Niemaszyk A., Langfort J., Gajda R. (2025). Effects of Normobaric Hypoxia of Varying Severity on Metabolic and Hormonal Responses Following Resistance Exercise in Men and Women. J. Clin. Med..

[33] Zani A., Dishi Y., Proverbio A.M. (2024). From oxygen shortage to neurocognitive challenges: Behavioral patterns and imaging insights. Front. Cogn..

[34] Dmytriv T.R., Duve K.V., Storey K.B., Lushchak V.I. (2024). Vicious cycle of oxidative stress and neuroinflammation in pathophysiology of chronic vascular encephalopathy. Front. Physiol..

[35] Coimbra-Costa D., Alva N., Duran M., Carbonell T., Rama R. (2017). Oxidative stress and apoptosis after acute respiratory hypoxia and reoxygenation in rat brain. Redox Biol..

[36] Turner C.E., Barker-Collo S.L., Connell C.J.W., Gant N. (2015). Acute hypoxic gas breathing severely impairs cognition and task learning in humans. Physiol. Behav..

[37] Damgaard V., Mariegaard J., Lindhardsen J.M., Ehrenreich H., Miskowiak K.W. (2023). Neuroprotective Effects of Moderate Hypoxia: A Systematic Review. Brain Sci..

[38] McMorris T., Hale B.J., Barwood M., Costello J., Corbett J. (2017). Effect of acute hypoxia on cognition: A systematic review and meta-regression analysis. Neurosci. Biobehav. Rev..

[39] Young J.M., Williams D.R., Thompson A.A.R. (2019). Thin Air, Thick Vessels: Historical and Current Perspectives on Hypoxic Pulmonary Hypertension. Front. Med..

[40] Chen X. (2019). Combined fractional anisotropy and subcortical volumetric abnormalities in healthy immigrants to high altitude: A longitudinal study. Hum. Brain Mapp..

[41] Li G. (2022). Chronic hypoxia leads to cognitive impairment by promoting HIF-2α-mediated ceramide catabolism and alpha-synuclein hyperphosphorylation. Cell Death Discov..

[42] Lei L. (2022). HIF-1α Causes LCMT1/PP2A Deficiency and Mediates Tau Hyperphosphorylation and Cognitive Dysfunction during Chronic Hypoxia. Int. J. Mol. Sci..

[43] Allsopp G.L., Addinsall A.B., Hoffmann S.M., Russell A.P., Wright C.R. (2022). Hormonal and metabolic responses of older adults to resistance training in normobaric hypoxia. Eur. J. Appl. Physiol..

[44] Seitzman B.A., Snyder A.Z., Leuthardt E.C., Shimony J.S. (2019). The State of Resting State Networks. Top. Magn. Reson. Imaging.

[45] Greicius M.D., Krasnow B., Reiss A.L., Menon V. (2003). Functional connectivity in the resting brain: A network analysis of the default mode hypothesis. Proc. Natl. Acad. Sci. USA.

[46] Menon V. (2023). 20 years of the default mode network: A review and synthesis. Neuron.

[47] Smallwood J., Bernhardt B.C., Leech R., Bzdok D., Jefferies E., Margulies D.S. (2021). The default mode network in congnition: A topographical perspective. Nat. Rev. Neurosci..

[48] Hughes C., Setton R., Mwilambwe-Tshilobo L., Baracchini G., Tuner G.R., Spreng N. (2024). Precision mapping of the default mode network reveals common and distinct (inter) activity for autobiographical memory and theory mind. J. Neurophysiol..

[49] Ezama L., Hernández-Cabrera J.A., Seoane S., Pereda E., Janssen N. (2021). Functional connectivity of the hippocampus and its subfields in resting-state networks. Eur. J. Neurosci..

[50] Barnett A.J., Reilly W., Dimsdale-Zucker H.R., Mizrak E., Reagh Z., Ranganath C. (2021). Intrinsic connectivity reveals functionally distinct cortico-hippocampal networks in the human brain. PLoS Biol..

[51] Danieli K., Guyon A., Bethus I. (2023). Episodic Memory formation: A review of complex Hippocampus input pathways. Prog. Neuro-Psychopharmacol. Biol. Psychiatry.

[52] Gordon E.M., Laumann T.O., Gilmore A.W., Newbold D.J., Greene D.J., Berg J.J., Ortega M., Hoyt-Drazen C., Gratton C., Sun H. (2017). Precision Functional Mapping of Individual Human Brains. Neuron.

[53] Uddin L.Q. (2016). Salience Network of the Human Brain.

[54] Andreano J.M., Touroutoglou A., Dickerson B.C., Barrett L.F. (2017). Resting connectivity between salience nodes predicts recognition memory. Soc. Cogn. Affect. Neurosci..

[55] Liang X., Zou Q., He Y., Yang Y. (2016). Topologically Reorganized Connectivity Architecture of Default-Mode, Executive-Control, and Salience Networks across Working Memory Task Loads. Cereb. Cortex.

[56] Fang X. (2016). Resting-State Coupling between Core Regions within the Central-Executive and Salience Networks Contributes to Working Memory Performance. Front. Behav. Neurosci..

[57] Machner B. (2022). Resting-State Functional Connectivity in the Dorsal Attention Network Relates to Behavioral Performance in Spatial Attention Tasks and May Show Task-Related Adaptation. Front. Hum. Neurosci..

[58] Bernard F., Lemee J.-M., Mazerand E., Leiber L.-M., Menei P., Ter Minassian A. (2020). The ventral attention network: The mirror of the language network in the right brain hemisphere. J. Anat..

[59] Vossel S., Geng J.J., Fink G.R. (2014). Dorsal and Ventral Attention Systems: Distinct Neural Circuits but Collaborative Roles. Neuroscientist.

[60] Dunne L., Opitz B. (2020). Attention control processes that prioritise task execution may come at the expense of incidental memory encoding. Brain Cogn..

[61] Aly M., Turk-Browne N.B. (2016). Attention Stabilizes Representations in the Human Hippocampus. Cereb. Cortex.

[62] van Ede F., Board A.G., Nobre A.C. (2020). Goal-directed and stimulus-driven selection of internal representations. Proc. Natl. Acad. Sci. USA.

[63] Lee M.H., Smyser C.D., Shimony J.S. (2013). Resting-State fMRI: A Review of Methods and Clinical Applications. Am. J. Neuroradiol..

[64] Kassab R., Alexandre F. (2018). Pattern separation in the hippocampus: Distinct circuits under different conditions. Brain Struct. Funct..

[65] Pena E., El Alam S., Siques P., Brito J. (2022). Oxidative Stress and Diseases Associated with High-Altitude Exposure. Antioxidants.

[66] Ramírez-delaCruz M., Bravo-Sánchez A., Sánchez-Infante J., Abián P., Abián-Vicén J. (2024). Effects of Acute Hypoxic Exposure in Simulated Altitude in Healthy Adults on Cognitive Performance: A Systematic Review and Meta-Analysis. Biology.

[67] Davranche K., Casini L., Arnal P.J., Rupp T., Perrey S., Verges S. (2016). Cognitive functions and cerebral oxygenation changes during acute and prolonged hypoxic exposure. Physiol. Behav..

[68] Critchley H.D. (2015). Slow Breathing and Hypoxic Challenge: Cardiorespiratory Consequences and Their Central Neural Substrates. PLoS ONE.

[69] ADAMSON M.M. (2012). Pilot Expertise and Hippocampal Size: Associations with Longitudinal Flight Simulator Performance. Aviat. Space Environ. Med..

[70] Chen X. (2020). Altered Default Mode Network Dynamics in Civil Aviation Pilots. Front. Neurosci..

[71] Cheng H., Sun G., Li M., Yin M., Chen H. (2019). Neuron loss and dysfunctionality in hippocampus explain aircraft noise induced working memory impairment: A resting-state fMRI study on military pilots. Biosci. Trends.

[72] Liu J. (2015). Changes in resting-state brain function of pilots after hypoxic exposure based on methods for fALFF and ReHo analysis. Med. J. Chin. Peoples Lib. Army.

[73] Xu K. (2023). Research on brain functions related to visual information processing and body coordination function of pilots based on the low-frequency amplitude method. Front. Hum. Neurosci..

[74] Annen J. (2021). Mapping the functional brain state of a world champion freediver in static dry apnea. Brain Struct. Funct..

[75] Song R., Tao G., Guo F., Ma H., Zhang J., Wang Y. (2023). The change of attention network functions and physiological adaptation during high-altitude hypoxia and reoxygenation. Physiol. Behav..

[76] Miskowiak K.W. (2024). Effects of cognitive training under hypoxia on cognitive proficiency and neuroplasticity in remitted patients with mood disorders and healthy individuals: ALTIBRAIN study protocol for a randomized controlled trial. Trials.

[77] Zhang G. (2024). Intermittent hypoxia training effectively protects against cognitive decline caused by acute hypoxia exposure. Pflüg. Arch.—Eur. J. Physiol..

[78] Wang L., Sang L.Q., Cui Y., Li P.Y., Qiao L., Wang Q.N., Zhao W.Q., Hu Q., Zhang N.J., Zhang Y. (2022). Effects of acute high-altitude exposure on working memory: A functional near-infrared spectroscopy study. Brain Behav..

[79] Zhang X. (2024). Neuroplasticity of visual brain network induced by hypoxia. Cereb. Cortex.

[80] Sharma P., Pandey P., Kumari P., Sharma N.K., Sharma N.K., Arya A. (2022). Introduction to High Altitude and Hypoxia. High Altitude Sickness—Solutions from Genomics, Proteomics and Antioxidant Interventions.

[81] Zhang W. (2024). Investigating Sea-Level Brain Predictors for Acute Mountain Sickness: A Multimodal MRI Study before and after High-Altitude Exposure. Am. J. Neuroradiol..

[82] Limmer M., Platen P. (2018). The influence of hypoxia and prolonged exercise on attentional performance at high and extreme altitudes: A pilot study. PLoS ONE.

[83] Li H.-X. (2019). Resting-state network complexity and magnitude changes in neonates with severe hypoxic ischemic encephalopathy. Neural Regen. Res..

[84] Wang Y. (2023). Changes of Functional Brain Network in Neonates with Different Degrees of Hypoxic-Ischemic Encephalopathy. Brain Connect..

[85] Jiang L. (2022). Alterations in motor functional connectivity in Neonatal Hypoxic Ischemic Encephalopathy. Brain Inj..

[86] Boerwinkle V.L. (2022). Association of network connectivity via resting state functional MRI with consciousness, mortality, and outcomes in neonatal acute brain injury. NeuroImage Clin..

[87] Spencer A.P.C., Goodfellow M., Chakkarapani E., Brooks J.C.W. (2024). Resting-state functional connectivity in children cooled for neonatal encephalopathy. Brain Commun..

[88] Jung W.-B. (2016). Neuroplasticity for spontaneous functional recovery after neonatal hypoxic ischemic brain injury in rats observed by functional MRI and diffusion tensor imaging. NeuroImage.

[89] Yuan H., Wang Y., Liu P.-F., Yue Y.-L., Guo J.-S., Wang Z.-C. (2019). Abnormal brain activity in rats with sustained hypobaric hypoxia exposure: A resting-state functional magnetic resonance imaging study. Chin. Med. J..

[90] Miller J. (2018). Lateralized hippocampal oscillations underlie distinct aspects of human spatial memory and navigation. Nat. Commun..

[91] Zhang X. (2023). Consistent differences in brain structure and functional connectivity in high-altitude native Tibetans and immigrants. Brain Imaging Behav..

[92] Zhang L. (2022). Hippocampal adaptation to high altitude: A neuroanatomic profile of hippocampal subfields in Tibetans and acclimatized Han Chinese residents. Front. Neuroanat..

[93] Li Y., Wang Y. (2022). Effects of Long-Term Exposure to High Altitude Hypoxia on Cognitive Function and Its Mechanism: A Narrative Review. Brain Sci..

[94] Su R. (2024). The effects of long-term high-altitude exposure on cognition: A meta-analysis. Neurosci. Biobehav. Rev..

[95] Yan X., Zhang J., Gong Q., Weng X. (2011). Prolonged high-altitude residence impacts verbal working memory: An fMRI study. Exp. Brain Res..

[96] Xin Z. (2020). Alteration in topological properties of brain functional network after 2-year high altitude exposure: A panel study. Brain Behav..

[97] Chen X. (2017). Cognitive and neuroimaging changes in healthy immigrants upon relocation to a high altitude: A panel study. Hum. Brain Mapp..

[98] Lehn H., Steffenach H.-A., van Strien N.M., Veltman D.J., Witter M.P., Håberg A.K. (2009). A Specific Role of the Human Hippocampus in Recall of Temporal Sequences. J. Neurosci..

[99] Kumaran D., Maguire E.A. (2007). Match–Mismatch Processes Underlie Human Hippocampal Responses to Associative Novelty. J. Neurosci..

[100] Maguire E.A., Frith C.D. (2003). Lateral Asymmetry in the Hippocampal Response to the Remoteness of Autobiographical Memories. J. Neurosci..

[101] Zhang X. (2022). Resting-State Neuronal Activity and Functional Connectivity Changes in the Visual Cortex after High Altitude Exposure: A Longitudinal Study. Brain Sci..

[102] Chen X. (2021). Altered resting-state networks may explain the executive impairment in young health immigrants into high-altitude area. Brain Imaging Behav..

[103] Zhang Y.Q., Zhang W., Liu J., Ji W. (2022). Effects of Chronic Hypoxic Environment on Cognitive Function and Neuroimaging Measures in a High-Altitude Population. Front. Aging Neurosci..

[104] Zhang J. (2013). Adaptive Modulation of Adult Brain Gray and White Matter to High Altitude: Structural MRI Studies. PLoS ONE.

[105] Zhang J. (2017). Alteration of Spontaneous Brain Activity After Hypoxia-Reoxygenation: A Resting-State fMRI Study. High Alt. Med. Biol..

[106] Zhong M., Zeng H., Wang D., Li J., Duan X., Li Y. (2022). Structure and activity alteration in adult highland residents’ cerebrum: Voxel-based morphometry and amplitude of low-frequency fluctuation study. Front. Neurosci..

[107] Zhang X. (2023). Brain Structural and Functional Alterations in Native Tibetans Living at High Altitude. Neuroscience.

[108] Chen J. (2016). Increased Intraregional Synchronized Neural Activity in Adult Brain after Prolonged Adaptation to High-Altitude Hypoxia: A Resting-State fMRI Study. High Alt. Med. Biol..

[109] Cao Y., Cao S., Ge R.-L., Bao H., Mou Y., Ji W. (2023). Brain-aging related protein expression and imaging characteristics of mice exposed to chronic hypoxia at high altitude. Front. Aging Neurosci..

[110] Cramer N.P. (2019). Neuronal and vascular deficits following chronic adaptation to high altitude. Exp. Neurol..

[111] Luo Q., Zhang J.-X., Huang S., Hu Y.-H., Wang H., Chen X. (2023). Effects of long-term exposure to high altitude on brain structure in healthy people: An MRI-based systematic review and meta-analysis. Front. Psychiatry..

[112] Veasey S.C., Rosen I.M. (2019). Obstructive Sleep Apnea in Adults. N. Engl. J. Med..

[113] Bao J. (2024). Elucidating the association of obstructive sleep apnea with brain structure and cognitive performance. BMC Psychiatry.

[114] Chang Y.-T. (2020). Functional connectivity in default mode network correlates with severity of hypoxemia in obstructive sleep apnea. Brain Behav..

[115] Kang D. (2020). Brain functional changes in tibetan with obstructive sleep apnea hypopnea syndrome: A resting state fMRI study. Medicine.

[116] Qin Z., Kang D., Feng X., Kong D., Wang F., Bao H. (2020). Resting-state functional magnetic resonance imaging of high altitude patients with obstructive sleep apnoea hypopnoea syndrome. Sci. Rep..

[117] Tung A., Takase L., Fornal C., Jacobs B. (2005). Effects of sleep deprivation and recovery sleep upon cell proliferation in adult rat dentate gyrus. Neuroscience.

[118] Li K. (2022). Dynamic regional homogeneity alterations and cognitive impairment in patients with moderate and severe obstructive sleep apnea. Front. Neurosci..

[119] Huang L. (2023). Abnormal dynamic functional connectivity in the hippocampal subregions of patients with untreated moderate-to-severe obstructive sleep apnea. Sleep Med..

[120] He Y., Shen J., Wang X., Wu Q., Liu J., Ji Y. (2022). Preliminary study on brain resting-state networks and cognitive impairments of patients with obstructive sleep apnea–hypopnea syndrome. BMC Neurol..

[121] Park H.R., Cha J., Joo E.Y., Kim H. (2022). Altered cerebrocerebellar functional connectivity in patients with obstructive sleep apnea and its association with cognitive function. Sleep.

[122] Shu Y. (2022). Inherent regional brain activity changes in male obstructive sleep apnea with mild cognitive impairment: A resting-state magnetic resonance study. Front. Aging Neurosci..

[123] Chen L.-T. (2017). Disrupted small-world brain functional network topology in male patients with severe obstructive sleep apnea revealed by resting-state fMRI. Neuropsychiatr. Dis. Treat..

[124] Peng D.-C., Dai X.-J., Gong H.-H., Li H.-J., Nie X., Zhang W. (2014). Altered intrinsic regional brain activity in male patients with severe obstructive sleep apnea: A resting-state functional magnetic resonance imaging study. Neuropsychiatr. Dis. Treat..

[125] Naismith S.L. (2020). Nocturnal Hypoxemia Is Associated with Altered Parahippocampal Functional Brain Connectivity in Older Adults at Risk for Dementia. J. Alzheimer’s Dis..

[126] Bai J. (2021). Altered Spontaneous Brain Activity Related to Neurologic and Sleep Dysfunction in Children With Obstructive Sleep Apnea Syndrome. Front. Neurosci..

[127] Bao H., He X., Wang F., Kang D. (2021). Study of Brain Structure and Function in Chronic Mountain Sickness Based on fMRI. Front. Neurol..

[128] Davenport P.W., Vovk A. (2009). Cortical and subcortical central neural pathways in respiratory sensations. Respir. Physiol. Neurobiol..

[129] Harper R.M. (2003). fMRI responses to cold pressor challenges in control and obstructive sleep apnea subjects. J. Appl. Physiol..

[130] Macey K.E. (2004). fMRI signal changes in response to forced expiratory loading in congenital central hypoventilation syndrome. J. Appl. Physiol..

[131] Guan L., Ge R., Ma S. (2025). Impact of hypoxia on the hippocampus: A review. Medicine.

[132] Yu J.J., Non A.L., Heinrich E.C., Gu W., Alcock J., Moya E.A., Lawrence E.S., Tift M.S., O’Brien K.A., Storz J.F. (2022). Time Domains of Hypoxia Responses and -Omics Insights. Front. Physiol..

[133] Howard R.S., Holmes P.A., Koutroumanidis M.A. (2011). Hypoxic-ischaemic brain injury. Pract. Neurol..

[134] Kolisnyk M. (2023). Predicting neurologic recovery after severe acute brain injury using resting-state networks. J. Neurol..

[135] Cole D.M., Smith S.M., Beckmann C.F. (2010). Advances and pitfalls in the analysis and interpretation of resting-state FMRI data. Front. Syst. Neurosci..

[136] Duncan N.W., Northoff G. (2013). Overview of potential procedural and participant-related confounds for neuroimaging of the resting state. J. Psychiatry Neurosci..

[137] Le L.N.N. (2023). Cortical oxygen extraction fraction using quantitative BOLD MRI and cerebral blood flow during vasodilation. Front. Physiol..

[138] Deckers P.T. (2022). Hemodynamic and metabolic changes during hypercapnia with normoxia and hyperoxia using pCASL and TRUST MRI in healthy adults. J. Cereb. Blood Flow Metab..

[139] Chen J.J., Gauthier C.J. (2021). The Role of Cerebrovascular-Reactivity Mapping in Functional MRI: Calibrated fMRI and Resting-State fMRI. Front. Physiol..

[140] Kurban D., Ivanov D., Kashyap S., Huber L., Liberman G., Poser B.A. (2022). Concurrent CBF and BOLD FMRI with dual-echo spiral simultaneous multi-slice acquisitions at 7T. NeuroImage.

[141] Cohen A.D., Nencka A.S., Lebel R.M., et Wang Y. (2017). Multiband multi-echo imaging of simultaneous oxygenation and flow timeseries for resting state connectivity. PLoS ONE.

[142] Cohen A.D., Nencka A.S., Wang Y. (2018). Multiband multi-echo simultaneous ASL/BOLD for task-induced functional MRI. PLoS ONE.

[143] Kang D., In M.H., Jo H.J., Halverson M., Meyer N.K. (2023). Improved Resting-State Functional MRI Using Multi-Echo Echo-Planar Imaging on a Compact 3T MRI Scanner with High-Performance Gradients. Sensors.

